# An Introduction to Automated Flow Cytometry Gating Tools and Their Implementation

**DOI:** 10.3389/fimmu.2015.00380

**Published:** 2015-07-27

**Authors:** Chris P. Verschoor, Alina Lelic, Jonathan L. Bramson, Dawn M. E. Bowdish

**Affiliations:** ^1^Department of Pathology and Molecular Medicine, McMaster Immunology Research Centre (MIRC), McMaster University, Hamilton, ON, Canada

**Keywords:** flow cytometry, automated analysis, gating, high-throughput, software, immunology

## Abstract

Current flow cytometry (FCM) reagents and instrumentation allow for the measurement of an unprecedented number of parameters for any given cell within a homogenous or heterogeneous population. While this provides a great deal of power for hypothesis testing, it also generates a vast amount of data, which is typically analyzed manually through a processing called “gating.” For large experiments, such as high-content screens, in which many parameters are measured, the time required for manual analysis as well as the technical variability inherent to manual gating can increase dramatically, even becoming prohibitive depending on the clinical or research goal. In the following article, we aim to provide the reader an overview of automated FCM analysis as well as an example of the implementation of FLOw Clustering without K, a tool that we consider accessible to researchers of all levels of computational expertise. In most cases, computational assistance methods are more reproducible and much faster than manual gating, and for some, also allow for the discovery of cellular populations that might not be expected or evident to the researcher. We urge any researcher who is planning or has previously performed large FCM experiments to consider implementing computational assistance into their analysis pipeline.

## Introduction

Recent advances in flow cytometry (FCM) have provided researchers in the fields of cellular and clinical immunology an incredible amount of leverage toward testing new hypotheses. These include improvements to reagents and instrumentation employed in traditional fluorescence-based FCM, allowing for the measurement of up to 20 parameters for any given cell (1), as well as the introduction of mass cytometry [CyTOF, reviewed in Ref. (2)], which can measure up to 34 parameters for any given cell. These technologies, while allowing for the discrimination of new and sometimes rare populations within a heterogeneous mixture of cells (akin to a needle in a haystack), also produce a tremendous amount of data, which is most commonly analyzed manually using proprietary software (for example, FlowJo)^1^. For many immunologists, the manual analysis of FCM data does not hinder productivity, given that most FCM experiments are performed on a small number (<40) of experimental units and involve less than a half dozen parameters. However, in the case of experiments that require the interrogation of more than 10 parameters in hundreds of experimental units, the manual analysis of FCM can become a significant expenditure of time. For example, our laboratory group has performed a number of studies on the frequency and phenotype of peripheral blood ­leukocytes (white blood cells) in elderly individuals, discriminating up to 16 cell surface and intracellular molecules (across multiple stains) in 130 to more than a 1000 individuals (3–7). For any one of these studies, the time required for FCM analysis alone was >15 h.

In the following review, we will provide an overview of the methods to automate FCM analysis computationally and describe one of the more accessible solutions available to researchers with little or no experience in computer programing. We urge any researcher who has conducted or is considering conducting a large-scale FCM study to evaluate these tools as well as many of the other exquisite techniques that are freely available to the scientific community.

## An Overview of Flow Cytometry

**Figure 1:**
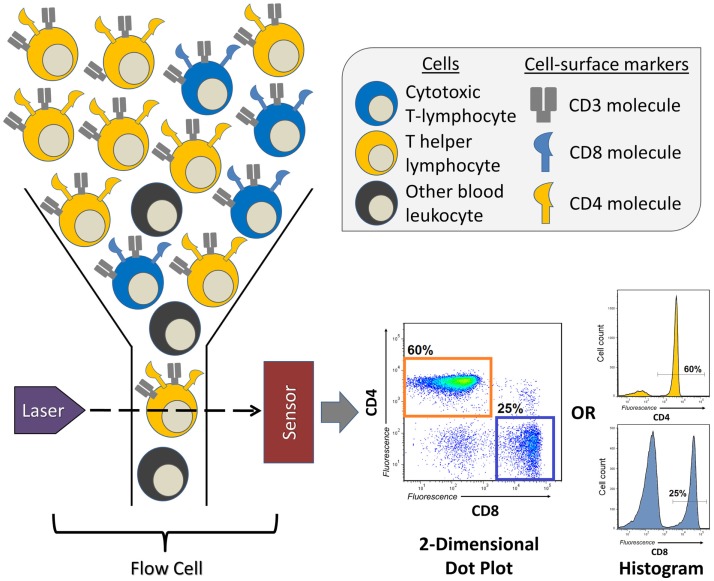
**A brief overview of a flow cytometry experiment identifying the proportions of T helper and cytotoxic T-lymphocytes in human peripheral blood**. Peripheral blood leukocytes are first stained with three antibodies conjugated to unique fluorescent dyes (not represented here), which will specifically bind the T-lymphocyte markers (antigens) CD3, CD4, or CD8. This sample of leukocytes is then transferred to the flow cytometer’s flow cell, which focuses the stream of leukocytes, allowing them to pass through the laser beam one at a time. The fluorescent dyes are then excited by the laser, and their emitted spectrum is detected by sensors, which digitize the information and visualizes it as two-dimensional dot plots, or one-dimensional histograms. The levels of CD3, CD4, and CD8 are recorded for every leukocyte that passes through the flow cell, therefore, allowing for the quantification of frequency of CD3^+^CD4^+^ T helper or CD3^+^CD8^+^ cytotoxic T-lymphocytes.

Invented in the 1960s, and first described in 1972 (8), FCM or fluorescence-activated cell sorting (FACS), as it was first called, has transformed a number of fields, most of which being cellular and clinical immunology. It allows for the quantification of either surface or intracellularly expressed molecules (also known as antigens) for a single cell within a larger population, doing so in a high-throughput fashion, providing measurements for hundreds to thousands of cells per second. FCM is particularly useful in situations in which the researcher wishes to discriminate between multiple different cell types within a heterogeneous population and measure their individual frequencies or the expression of a specific molecule of interest (Figure 1). For example, FCM is often used to measure the frequency of two major T-lymphocyte populations in the peripheral blood, T helper cells and cytotoxic T-cells, which is easily accomplished by distinguishing leukocytes according to the expression of the cell-surface molecules CD4 (T helper cell) and CD8 (cytotoxic T-cell) (Figure 1). In many clinical studies, FCM has proved to be indispensable, allowing for the measurement of distinct, sometimes rare populations of cells that are indicative of the progression of disease (9) or the success of therapeutic intervention (10).

### Gating

**Figure 2:**
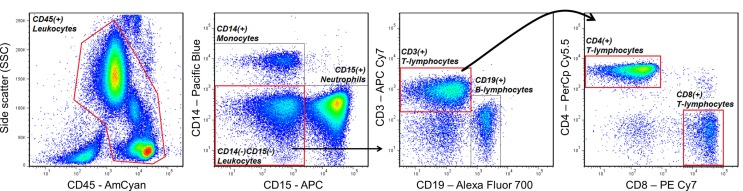
**An example of a gating strategy used to identify CD4^+^ T helper and CD8^+^ cytotoxic T-lymphocytes in human peripheral blood**. Antibodies that specifically recognize the cell-surface markers CD45, CD14, CD15, CD3, CD19, CD4, and CD8, and conjugated to the fluorescent dyes AmCyan, Pacific Blue, Allophycocyanin (APC), APC Cy7, Alexa Fluor 700, PerCp Cy5.5, and PE Cy7, respectively, were used to sequentially identify T-lymphocytes expressing CD45, CD3, and CD4 or CD8, while excluding cells expressing CD14 (monocytes), CD15 (neutrophils), or CD19 (B-lymphocytes).

One of the most basic principles of FCM analysis is “gating,” which is the sequential identification and refinement of a cellular population of interest using a panel of molecules (also known as markers) that are visualized by fluorescence in a unique emission spectrum. For example, if a researcher is interested in quantifying the proportions of CD4 expressing T helper cells and CD8 expressing cytotoxic T-cells in peripheral blood, he/she may use a combination of antibodies (which specifically recognize the marker of interest) conjugated to unique fluorescent dyes that will accurately identify these cells, while discriminating other cell types that are not of interest. In the example of Figure 2, the cells of interest that are being selected (gated) express CD45 (a pan-leukocyte marker), CD3 (a marker specific to mainly T-lymphocytes), and CD4, or CD8. Cells that are not of interest and will be “gated out” also express CD45, but uniquely express CD14 (commonly expressed on monocytes), CD15 (commonly expressed on neutrophils), or CD19 (commonly expressed on B-lymphocytes). Thus, according to the fluorescence of dyes conjugated to antibodies recognizing each marker, the researcher will be able to identify T helper cells and cytotoxic T-cells, which may also be labeled CD45^+^CD14^−^CD15^−^CD19^−^CD3^+^CD4^+^CD8^−^ and CD45^+^CD14^−^CD15^−^CD19^−^CD3^+^CD4^−^CD8^+^, respectively. Additionally, based on the level of fluorescence for a given marker, the researcher can also measure the degree to which that molecule is being expressed by the cell of interest.

## Computational Assistance for FCM Analysis

### Why you should consider using computational assistance methods

One of the three primary reasons that researchers should consider implementing computational assistance for their FCM analysis is speed. As previously mentioned, the time it takes to manually analyze an FCM experiment is dependent on the number of experimental units to process as well as the number of markers (also known as parameters) needed to gate. This can increase the analysis time dramatically if the gating strategy is particularly complex. Depending on the particular software package used, the use of computational assistance can reduce FCM analysis time from hours to minutes (11).

The inherent subjectivity of manual analysis should be considered another major reason for the use of computational assistance for FCM analysis. Since manual analysis requires the user to specify which cells to gate on (often called a “gating strategy”), additional technical variation will naturally arise due to the inability of humans to accurately reproduce this strategy within and across FCM experiments. The amount of technical variability related to human subjectivity, not surprisingly, is increased if more than one user is tasked to perform the FCM analysis, and has been estimated to be as high as 78% (12, 13). Computational assistance not only removes the necessity for multiple individuals to take part in FCM analysis but also compared to manual gating, many software packages have been shown to be vastly superior with regards to the variability observed due to the gating procedure, even when applied to experiments performed with heterogeneous protocols and reagents (13–15).

Finally, another great benefit to the use of computational assistance for FCM analysis is the potential to discover new, biologically relevant cellular populations that were not initially considered by the researcher. As previously discussed, the basis of manual analysis is a gating strategy that allows one to capture data for particular cellular populations of interest. Computational methods are efficient “discovery” tools because they do not rely on any particular gating strategy or guidance from the end-user to identify cellular populations in FCM experiments. Instead, they apply mathematical algorithms that are able to detect trends within an entire FCM dataset, which are inferred to be *bona fide* cellular populations. Hence, populations that were not specified in a given gating strategy may be identified by computational analysis. That being said, upon discovering a new cellular population, it is still necessary for researchers to validate their finding biologically. Most computational methods are unable to distinguish between true fluorescence due to marker expression and artifactual fluorescence due to cellular autofluorescence or fluorescent spillover (16), and some will simply overestimate the number of cellular populations without guidance from the end-user (17).

### An overview of some of the procedures used by computational assistance software packages

**Figure 3:**
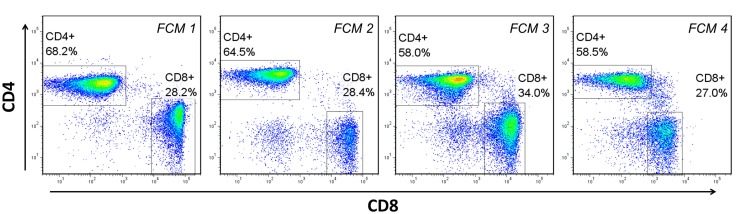
**Biological and technical variability between flow cytometry (FCM) experiments**. Four separate experiments, performed on different days with peripheral blood mononuclear cells from at least two different donors demonstrates the variability in the fluorescent staining patterns that is common in FCM analysis. FCM 1–3 represent experiments performed on different donors over different days. FCM 3 and 4 represent the same donor analyzed on different days.

Dozens of freely available software packages have been reported [see Ref. (11, 18)], and can be categorized anywhere from being completely automated without the need for guidance from the end-user, to being partially automated and requiring a great deal of guidance and adjustment (tuning) in order to complete its task. The basis of these packages, which allow them to adapt to experimental environments that often include a great deal of technical and biological variability (Figure 3), is the algorithms and mathematical procedures upon which they are built. Below is a brief summary of these approaches, including inherent advantages and disadvantages.

According to Bashashati and Brinkman (18), there are five distinct requirements for an automated gating procedure: (1) computational efficiency, (2) the ability to identify a cellular population regardless of shape, (3) robustness toward different antigen/marker densities and expression patterns, (4) the ability to determine the true population number accurately, and (5) the ability to detect and account for outliers. All of these requirements essentially surround the capacity of the software to correctly identify clusters or groups of data points, which are assumed to be *bona fide* cellular populations. While there are many approaches to do this, the most common use clustering algorithms, with k-means clustering being the most popular, and model-based algorithms.

k-Means clustering is an iterative process in which “k” number of clusters are defined (often by the user) and the center of each cluster (initially assigned randomly) is refined until each cluster encompasses a unique set of data points; in other words, each FCM data point ends up being closest to only one cluster center. Naturally, the clusters tend to center in areas of density, which are assumed to be cellular populations (19). Major limitations of conventional k-means clustering with regards to automated FCM analysis is that the user must specify “k,” the number of clusters needed to be identified, and it is restricted to identifying spherically shaped populations (19). However, k-means is also a relatively fast procedure, which provides an important advantage over other automated gating algorithms. Some software packages [for example, flowMeans (20)] attempt to circumvent the drawback of *a priori* cluster specification by first computationally identifying the maximal number of clusters in a given FCM dataset, then iteratively collapsing that number by merging those clusters that significantly overlap.

As an alternative to clustering approaches, such as k-means, model-based approaches are attractive given that they are robust to the shape of cellular populations and do not require *a priori* input as does k-means clustering. However, these benefits come at a computational cost, and therefore, model-based approaches can be very time consuming (21). The most common approach is Gaussian, which requires FCM fluorescence data to follow a normal distribution, while others, such as t, skew-t, and uniform, offer more flexibility in this regard (18, 20). Some software packages use a combinational approach including k-means clustering and model-based algorithms to maximize efficiency and accuracy [for example, flowPeaks (22)], while others include hierarchical clustering in their combined approach [for example, Citrus (23) and Spade (24)].

**Figure 4:**
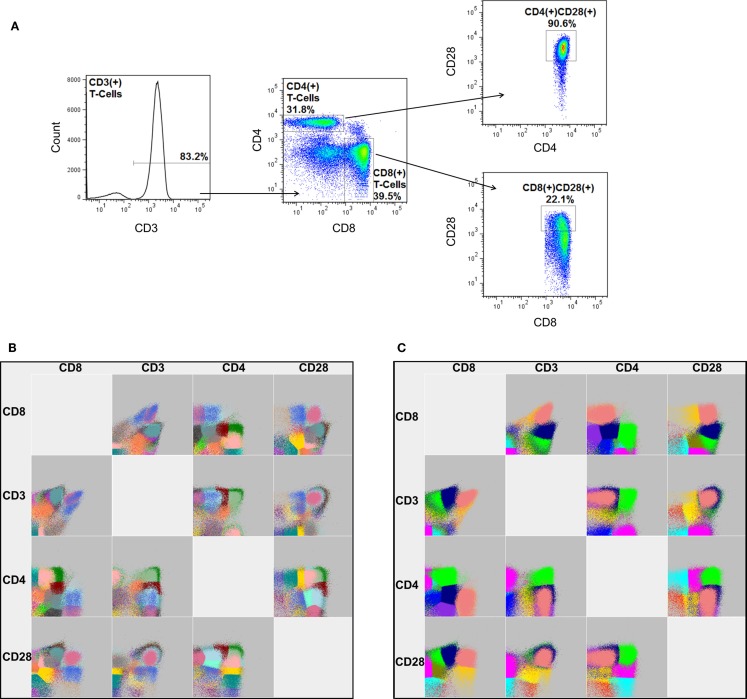
**A graphical comparison of cellular populations identified by manual gating and FLOCK**. **(A)** The gating strategy for manual analysis to sequentially identify the proportion of CD4 and CD8 expressing CD3^+^ lymphocytes, as well as the frequency of those that also express CD28. Note: the percentages shown represent the frequency of cells relative to the parent gate. FLOCK analysis was performed in parallel under **(B)** completely automated (no guidance) conditions as well as with **(C)** manual adjustment to the analysis parameters (bin = 30, density = 25). Each color represents a different cellular population as determined by FLOCK. Data employed have been previously published using a different analysis by Lelic et al. (26).

One of the most recently published software packages, flowDensity (25), offers a different approach. Instead of using clustering or model-based approaches to identify cellular populations, the software employs a manual gating strategy. This approach sacrifices the discovery aspect of automated FCM analysis, but benefits greatly in computational efficiency and the ability to accurately measure rare cellular populations, since they are effectively pre-defined by the end-user. Unlike many other packages, this software, as well as that of Feher and colleagues (16), can also make use of fluorescent-minus one (FMO) controls. An FMO is an important control for manual gating analysis in which a replicate FCM experiment is processed that includes all but one of the conjugated antibodies in the staining cocktail. Hence, the FMO provides the end-user an empirically derived fluorescence cut-off for the missing antibody’s respective dye. Software packages that incorporate FMO controls are expected to offer enhanced accuracy with regards to the measurement of rare cellular populations or those that are defined by markers that present as a continuous expression pattern (i.e., a smear, as in CD28 in Figure 4A, right panels).

As alluded to above, the approach of a given software package can have a major effect on its overall performance. To shed light on this issue in a standardized and unbiased manner, the flow cytometry: critical assessment of population identification methods (FlowCap) consortium was formed (11).

### Flow cytometry: Critical assessment of population identification methods

The FlowCap consortium represents leaders in the areas of mathematics, statistics, biology, and software development with the primary goal to objectively test and compare FCM computational assistance software and provide guidance on the best use of the available algorithms. To this date, the consortium has performed two competitions: FlowCap I, in which software packages were compared against manual gating under various levels of adjustment and tuning by the end-user, and FlowCap II, in which the ability of the software to define cellular populations that could then be used to stratify biological samples was tested. The results of these competitions can be found in Aghaeepour et al. (11).

For the FlowCap I competition, 14 software packages were employed to analyze 5 different FCM datasets submitted by experts in the fields of medicine and immunology, and their results compared against those derived from manual gating. Scores were presented using an *f*-score, where a score of 1 indicates that a particular software package perfectly mimics manual gating. In the first challenge, software packages were tested without any user guidance. From this, seven packages were observed to have an *f*-score of 0.80–0.90, the top four of which being ADICyt (commercially available from Adinis Ltd., Slovakia), flowMeans (20), FLAME (27), and FLOw Clustering without K (FLOCK) (28). Interestingly, manual tuning or adjustment of software analysis settings by the end-user only marginally improved the *f*-score of each package tested. However, if the number of identified populations desired by the end-user were used as guidance, the *f*-scores improved dramatically, with five algorithms ending up with scores of 0.90 or greater. As before, this not only included ADIcyt, FlowMeans, and FLOCK but also SamSpectral (29) and TCLUST (30). The overall conclusion from this challenge was that although some packages performed better than others, most performed well and this was largely dependent on the dataset analyzed. Furthermore, it was also observed that combining the results of multiple software packages (referred to as ensembl clustering) consistently performed best for all challenges, as high as 0.97, in fact.

For the second FlowCap competition, a series of software packages were tested for their ability to stratify a cohort of individuals into different classification groups based on their FCM data. This differs from the first challenge in that the particular cellular populations identified were not of interest, rather the software’s ability to identify populations that are highly correlated to a classification group (i.e., diseased or not diseased). The results from this challenge indicate that most packages are very good predictors of sample classification, and, in some cases, the software was perfect in its prediction. The results imply that using computational assistance will likely be an ideal fit for a clinical laboratory that relies on FCM for the diagnosis of patient health. However, the authors stress that the predictive capacity of these software packages is highly dependent on the nature of classification. In other words, for diseases in which FCM markers are not useful for diagnosis, computational assistance will provide little to no benefit.

## Implementation of Automated FCM Analysis for Immunologists

A major roadblock to the widespread implementation of automated FCM gating approaches is the perception by the scientific community that a great deal of technical expertise is required to operate them (31). While this is true for some software packages, many exist that can be easily and quickly employed by any researcher with access to the internet.

Flow cytometry analysis software can be broadly grouped into two areas: those driven by graphical-user interfaces (GUIs), which can be controlled by mouse and simple keyboard commands, and command-line driven modules, which require at least some computer programing language expertise. The former are commonly run from online servers or as stand-alone software, while the latter most commonly require proficiency in the programing language R^2^, an open-source and free-to-use software environment that has applications for a myriad of analyses in physical, biological, and social sciences. For those who are interested in learning R, there are a number of free-to-use, interactive online courses that can provide most individuals with enough proficiency to operate FCM analysis packages programed in this language; for example, Datacamp^3^ or Code School^4^.

For those who have not employed computation assistance for their FCM analyses, we will briefly introduce FLOCK (28), a software package that is easily accessible by most researchers. Using a small FCM dataset, we will compare the results from FLOCK using guidance free and tuned settings to manual gating as a frame of reference.

### FLOw clustering without K

FLOw Clustering without K was chosen because of its excellent performance in the FlowCap challenges (11) and represents an automated FCM analysis package that does not require any computer programing expertise, instead running from the publically available Immunology Database and Analysis Portal (Immport) (32) from the National Institute of Allergy and Infectious Diseases (NIAID). Furthermore, FLOCK does not require any manual adjustment to operate, although user adjustments are possible. FLOCK’s approach to identify cellular populations in an FCM experiment is grid-based partitioning of the data followed by density distribution analysis. In density distribution analysis, dense clusters of cells are identified and assigned a label at their center (known as a centroid), which can then be used to identify those same cellular clusters in other FCM experiments. Consequently, it is these two steps that users are able to manually adjust in order to refine their results; first, the number of bins (6–30), which specifies the degree to which the FCM environment is partitioned, and second, the density threshold cut-off (6–100), which modulates how the software determines whether two adjacent cellular clusters are one in the same or separate entities. There is also an automated mode where these two parameters are determined by the algorithm empirically. Many FCM results can be compared to each other after a canonical set of centroids is chosen by the user.

A major advantage of using FLOCK is that even though the user is able to manually tune the results, no guidance by way of a gating strategy is required. Hence, FLOCK can be very useful for identifying cellular populations that the researcher was not initially intending to measure. Another major advantage is its ease of implantation. A user can register with ImmPort, upload their FCM dataset (as FCS or text format), and perform FLOCK analysis in less than thirty minutes. Furthermore, the time to analyze a single FCM experiment file is trivial (minutes, for most experiments). A disadvantage of FLOCK, as shown in our example below, is that without manual tuning, FLOCK has a tendency to overestimate the number of cellular populations in a given FCM dataset. This, of course, is dependent on the particular dataset, which can vary greatly with regards to its heterogeneity and the number of parameters measured.

### Implementing FLOCK for automated FCM dataset analysis

To provide an example of FLOCK analysis, we analyzed a subset of FCM experiments from a previously published study (26). In this study, peripheral blood mononuclear cells from individuals aged 19–85 years old and acutely infected with West Nile virus (WNV) and chronically infected with Epstein–Barr virus (EBV) were tested for their ability to respond to peptides derived from these viruses. For the current analysis, a subset of 42 individuals were randomly chosen and the frequency of CD3^+^, CD3^+^CD4^+^, CD3^+^CD8^+^, CD3^+^CD4^+^CD28^+^, and CD3^+^CD8^+^CD28^+^ lymphocytes were measured by manual gating (Figure 4A) and FLOCK, under completely automated (Figure 4B) as well as manually tuned (Figure 4C) settings. Traditionally, the presence of these four markers on lymphocytes are discriminated as either expressed (+) or not expressed (−). Manual gating was performed using FlowJo, and simple comparison by Spearman’s rank correlation was performed to judge the performance of FLOCK.

Under completely automated settings FLOCK performed very quickly as compared to manual gating (23 min as compared to ~1 h), and as expected, the software also estimated more cellular populations than expected, 30 (Table 1). It should be noted that this includes a number of populations that would not be considered in the manual gating strategy (which is designed to measure only five populations), and includes cells that do not express CD3 or CD28, and those that express neither CD4 nor CD8. However, in addition to the conventional expression patterns for these five markers, either expressed (+) or not expressed (−), FLOCK also specifies “low” expressing populations (i.e., CD3^lo^) for each marker. This dramatically increases the number of populations identified. Although it is possible that a “low” expression pattern for CD3, CD4, CD8, or CD28 expressing lymphocytes is biologically relevant as opposed to an artifact of the FLOCK analysis, it is less likely that all 30 cellular populations are biologically and functionally distinct.

**Table 1:** **Cellular populations identified in our sample FCM dataset by manual gating and analysis by FLOCK**.

Manual gating	FLOCK (completely automated)	FLOCK (manual tuning)
CD3^+ a^	CD3^−^CD4^lo^CD8^−^CD28^lo^	CD3^−^CD4^lo^CD8^−^CD28^−^
CD3^+^CD4^+ b^	CD3^−^CD4^−^CD8^−^CD28^lo^	CD3^lo^CD4^lo^CD8^−^CD28^lo a,b^
CD3^+^CD4^+^CD28^+ c^	CD3^+^CD4^+^CD8^lo^CD28^+^	CD3^+^CD4^lo^CD8^−^CD28^+ a,b,c^
CD3^+^CD8^+ d^	CD3^−^CD4^lo^CD8^−^CD28^lo^	CD3^−^CD4^lo^CD8^−^CD28^lo^
CD3^+^CD8^+^CD28^+ e^	CD3^lo^CD4^−^CD8^−^CD28^lo^	CD3^lo^CD4^−^CD8^+^CD28^lo a,d^
	CD3^−^CD4^+^CD8^−^CD28^−^	CD3^+^CD4^−^CD8^lo^CD28^+ a,d^
	CD3^−^CD4^+^CD8^−^CD28^lo^	CD3^−^CD4^+^CD8^−^CD28^lo^
	CD3^−^CD4^+^CD8^−^CD28^lo^	CD3^−^CD4^+^CD8^−^CD28^−^
	CD3^+^CD4^lo^CD8^−^CD28^+^	CD3^−^CD4^+^CD8^−^CD28^lo^
	CD3^+^CD4^−^CD8^lo^CD28^+^	CD3^+^CD4^+^CD8^lo^CD28^+ a,b,c^
	CD3^+^CD4^lo^CD8^lo^CD28^+^	CD3^+^CD4^lo^CD8^lo^CD28^+ a^
	CD3^−^CD4^lo^CD8^−^CD28^lo^	CD3^+^CD4^−^CD8^+^CD28^lo a,d,e^
	CD3^−^CD4^−^CD8^−^CD28^−^	
	CD3^lo^CD4^lo^CD8^−^CD28^lo^	
	CD3^+^CD4^+^CD8^−^CD28^+^	
	CD3^+^CD4^−^CD8^lo^CD28^+^	
	CD3^−^CD4^+^CD8^−^CD28^−^	
	CD3^+^CD4^lo^CD8^lo^CD28^lo^	
	CD3^lo^CD4^lo^CD8^−^CD28^lo^	
	CD3^−^CD4^+^CD8^−^CD28^lo^	
	CD3^−^CD4^+^CD8^−^CD28^−^	
	CD3^+^CD4^lo^CD8^lo^CD28^+^	
	CD3^+^CD4^lo^CD8^lo^CD28^+^	
	CD3^−^CD4^+^CD8^−^CD28^lo^	
	CD3^−^CD4^lo^CD8^lo^CD28^lo^	
	CD3^+^CD4^lo^CD8^+^CD28^lo^	
	CD3^+^CD4^lo^CD8^+^CD28^+^	
	CD3^+^CD4^−^CD8^+^CD28^lo^	
	CD3^lo^CD4^−^CD8^+^CD28^−^	
	CD3^lo^CD4^−^CD8^+^CD28^lo^	

*Populations in the manual gating and FLOCK (manual tuning) groups with matching superscripts were combined within group and used to compare analysis methods*.

*Data employed have been previously published using a different analysis by Lelic et al. (26)*.

To reduce the resulting cellular populations identified to a number that is more likely to be biologically relevant, we adjusted the analysis parameters for FLOCK. This was determined on a fairly arbitrary basis, increasing the “bin” and “density” values until the number of cellular populations identified were no more than three times what is specified by our manual gating strategy. Using a bin value of 30 and a density value of 15, FLOCK identified 12 populations, which included “+,” “−,” and “lo” designations (Table 1). Further inspection of these populations indicated that as compared to the design of our manual gating strategy, FLOCK perceived the following: CD3^+^ cells included those labeled as CD3^+^ or CD3^lo^; CD4^+^ cells included those labeled as CD4^lo^CD8^−^ and CD4^+^CD8^lo^; CD8^+^ cells included those labeled as CD8^lo^CD4^−^ and CD8^+^CD4^−^; and CD28^+^ cells were either CD28^lo^ or CD28^+^, and no CD28^−^ cells were identified (Table 1). When the frequencies of those populations were combined into groups matching that in our manual gating strategy, and compared to our manual analysis using Spearman’s rank correlation, we found that the results from FLOCK are very similar. The ρ correlation for the CD3^+^, CD3^+^CD4^+^, CD3^+^CD4^+^CD28^+^, and CD3^+^CD8^+^ populations were 0.97, 0.96, 0.93, and 0.92, respectively, and significant at *p* < 0.0001 (Table 2). FLOCK estimated a higher frequency of cells in the CD3^+^CD8^+^CD28^+^ population, but was still comparable and significant (ρ = 0.62, *p* < 0.0001, Table 2).

**Table 2:** **Summary statistics and correlations of a paralleled analysis by manual gating and FLOCK (manually tuned)**.

	CD3^**+**^		CD4^**+**^		CD4^**+**^CD28^**+**^		CD8^**+**^		CD8^**+**^CD28^**+**^	
	Manual	Flock	Manual	Flock	Manual	Flock	Manual	Flock	Manual	Flock
Average	84.2	78.1	52.9	50.3	49.6	48.7	21.9	24.0	9.0	16.2
SE	1.4	1.7	2.7	2.2	2.7	2.4	1.6	1.5	0.7	1.1
25th Percentile	79.6	74.7	45.3	42.6	41.8	41.4	14.6	18.1	6.0	10.1
75th Percentile	91.3	85.7	63.5	60.9	59.7	58.5	26.4	27.0	10.8	20.3
Spearman’s ρ*	0.97		0.96		0.93		0.92		0.62	

*An FCM dataset of peripheral blood mononuclear cells from 42 individuals was analyzed to identify the proportion (relative to the total lymphoid population) of CD4 and CD8 expressing CD3^+^ lymphocytes, as well as the frequency of those that also express CD28. Data employed have been previously published using a different analysis by Lelic et al. (26)*.

*Significance of all Spearman’s rank correlations was *p* < 0.0001.

This brief comparison demonstrates the power of using FLOCK, namely the ability to identify cellular populations that were not specified in the manual gating strategy, the reduced time expenditure, and the high level of comparability to the results determined by manual analysis. The relatively lower correlation for the CD3^+^CD8^+^CD28^+^ population indicates that a software package may not be equally comparable to manual gating for all populations measured, and suggests that significant tuning of the analysis parameters may be required before a researcher is confident in the final analysis performed.

## Conclusion

In conclusion, we have provided an overview of automated FCM analysis as well as its advantages and disadvantages as compared to manual gating. There are numerous software packages to choose from, all of which differing slightly in the benefits they can provide to a given FCM dataset, as well as the technical expertise required to operate them. In our example we describe the results from FLOCK, a software package requiring little to no computing programing experience, and demonstrate the power of automated gating to improve the time required FCM analysis. Furthermore, our results from FLOCK demonstrate the potential for computational assistance to discover new, not previously considered cellular populations in a given FCM dataset. We urge any researcher who is planning or has previously performed large FCM experiments to consider implementing computational assistance into their analysis pipeline.

## Conflict of Interest Statement

The authors declare that the research was conducted in the absence of any commercial or financial relationships that could be construed as a potential conflict of interest.

## Footnotes

^1^www.flowjo.com

^2^http://cran.r-project.org/

^3^https://www.datacamp.com

^4^tryr.codeschool.com
